# The Views of Healthcare Professionals, Drug Developers and Regulators on Information about Older People Needed for Rational Drug Prescription

**DOI:** 10.1371/journal.pone.0072060

**Published:** 2013-08-16

**Authors:** Erna Beers, Toine C. G. Egberts, Hubert G. M. Leufkens, Paul A. F. Jansen

**Affiliations:** 1 Department of Geriatric Medicine and Expertise Centre Pharmacotherapy in Old Persons (EPHOR), University Medical Center Utrecht, Utrecht, The Netherlands; 2 Departments of Pharmacoepidemiology & Clinical Pharmacology, Utrecht Institute of Pharmaceutical Sciences (UIPS), Utrecht University, Utrecht, The Netherlands; 3 Departments of Clinical Pharmacy, University Medical Center Utrecht, Utrecht, The Netherlands; 4 Dutch Medicines Evaluation Board, The Hague, The Netherlands; Sapienza University of Rome, Italy

## Abstract

**Background:**

The ICH E7 guideline intends to improve the knowledge about medicines in geriatric patients. As a legislative document, it might not reflect the needs of healthcare professionals. This study investigated what information healthcare professionals, regulatory agencies and pharmaceutical industries consider necessary for rational drug prescribing to older individuals.

**Methods and Findings:**

A 29-item-questionnaire was composed, considering the representation in trials, pharmacokinetics, efficacy, safety, and convenience of use in older individuals, with space for additions. Forty-three European professionals with an interest in medication for older individuals were included. In order to investigate their relevance, five items were included in a second questionnaire, with 11 control items. Median scores, differences between clinical and non-clinical respondents and response consistency were analysed. Consistency was present in 10 control items. Therefore, all items of the first questionnaire and the five additional items were analysed. Thirty-seven (86%) respondents returned the first questionnaire; 31/37 (84%) the second. Information about age-related differences in adverse events, locomotor effects, drug-disease interactions, dosing instructions, and information about the proportion of included 65+ patients was considered necessary by most respondents. Clinicians considered information significantly more important than the non-clinical respondents about the inclusion of 75+, time-until-benefit in older people, anticholinergic effects, drug-disease interactions, and convenience of use. Main study limitations are the focus on information for daily practice, while the ICH E7 guideline is a legislative document focused on market approval of a new medicine. Also, a questionnaire with a Likert scale has its limitations; this was addressed by providing space for comments.

**Conclusions:**

This study reveals that items considered necessary are currently not included in the ICH E7 guideline. Also, clinicians’ and non-clinicians’ opinions differed significantly in 15% of the items. Therefore, all stakeholders should collaborate to improve the availability of information for the rational prescribing to older individuals.

## Introduction

National guidelines, such as the British National Formulary and the Physician’s Desk Reference, provide healthcare professionals with information about the rational prescribing of medicines. This information is typically based on the summary of product characteristics (SmPC) or product labelling. The SmPC is publicly available and provides information about the indication, dosing, warnings, and other basic features of medicines, and is intended as the official source of information for healthcare professionals for the effective and safe prescription of medicines [1]. The information in the SmPC as well as in the product labelling is derived from the pre-authorisation dossier.

Since in the pre-authorisation phase older people are often excluded from clinical trials [2]–[6], the International Conference on Harmonisation of Technical Requirements for Registration of Pharmaceuticals for Human Use (ICH), a committee of the drug regulatory authorities and the pharmaceutical industry of Europe, Japan, and the United States, developed a guideline for studies involving older individuals, focusing, from a legislative point of view, on what investigations should be carried out in older people, and what information should be reported in the pre-authorisation dossier of a new medicinal product [7]. Even though the guideline is not mandatory, a sponsor or pharmaceutical industry has to provide authorities with convincing reasons why it is not following these recommendations. This ICH E7 guideline, adopted in 1994, has been updated by the questions and answers document in 2010 [8].

The ICH E7 guideline is a legislative document [9]. Consequently, it might not reflect the needs of healthcare professionals in clinical practice. Therefore, the aim of this study is to investigate the opinions of clinical and non-clinical healthcare professionals about what information regarding older individuals should be available to facilitate rational prescribing by healthcare professionals before a medicine is approved by regulatory authorities and thereby evaluating the ICH E7 guideline.

## Methods

### Subjects

Geriatricians, nursing home physicians, internists, pharmacists, ethicists, regulators, as well as physicians, pharmacologists and pharmacists from the pharmaceutical industry with a professional interest in medication for older individuals were selected, with the intention of creating a group of at least 30 respondents. They were selected from several working groups on the basis of their professional activities: the European Academy for Medicine of Ageing (EAMA) network, in principle two people per country; the PREDICT consortium, in principle two people per country; the Committee for Medicinal Products for Human Use (CHMP) and the European Medicines Agency (EMA), if they had experience in evaluating the pre-authorisation dossiers of medicines for older individuals; and the Geriatrics Working Party of the European Forum on Good Clinical Practice (EFGCP). Eligible members had to be accessible via e-mail; 75 professionals were invited, by email, to participate in the study.

### Questionnaire

The respondents that gave written consent were asked to complete a questionnaire on the information needed to prescribe medications effectively and safely for older patients. The questionnaire contained 29 items (file S1) and was based on both the ICH E7 criteria [7] (16 items) and the questions and answers (Q&A) document [8] drawn up by the ICH (2 additional items), and the checklist of the Dutch formulary on prescribing to older patients [10] (11 additional items) (Table 1). The Q&A document is supplementary to the ICH E7 guideline and intends to clarify key issues [8]. The Dutch formulary had been developed on the basis of a Delphi study involving 63 Dutch medical and pharmacological experts.

**Table 1:** Themes and items used in the questionnaires and the sources of the items [7], [8], [10].

REPRESENTATION OF THE AGED	Source
Inclusion of patients >65 years in phase III studies	1
Inclusion of patients >75 years in phase III studies	1
For drugs used in diseases not unique for, but present in, old persons: inclusion of at least 100 patients >65 years in the phase III studies	1
For drugs used in diseases characteristically associated with aging (e.g., Alzheimer's disease): the majority of the clinical databaseconsists of geriatric patients	1
No exclusion of patients on the basis of an upper age cut-off	1
No exclusion based on concomitant medical conditions common in old persons (e.g., cardiovascular disease, diabetes, dementia)	1
No exclusion based on concomitant treatment with drugs commonly prescribed for old persons	2
The post-marketing data collection in geriatric patients is specified in the Risk Management Plan	2
How many subjects were included in the clinical program, who were not able to sign informed consent form themselves*	4
**PHARMACOKINETICS**	
A single-dose pharmacokinetic study in young versus old persons	1
A multiple-dose pharmacokinetic study in young versus old persons, if there are age-related differences in pharmacokinetics	1
The extent of drug accumulation in old persons	3
The extent of renal clearance of the active substances (i.e. parent compound and/or metabolites) in old persons	1, 3
The extent of hepatic clearance of the active substances (i.e. parent compound and/or metabolites) in old persons	1
The therapeutic dose range of the drug	1, 3
The extent of metabolism via or effects on specified CYP450 enzymes	1, 3
Potential drug-drug interactions, if the therapeutic range of the drug or likely concomitant drugs is narrow and the likelihoodof the concomitant therapy is great	1, 3
**EFFICACY**	
Age-related differences in efficacy	1, 3
Age-related differences in dose-response	1
If the medicinal product is indicated for a chronic condition: time until benefit in old persons	3
Information should be available about cost-effectiveness in older persons*	4
**SAFETY**	
Age-related differences in adverse events	1
Potential anticholinergic effects (e.g., cognitive decline, delirium, blurred vision, urine retention)	3
Potential sedative effects	3
Potential orthostatic effects	3
Potential effects on the locomotor system (e.g., decline of mobility, increased incidence of falls)	3
Potential cardiovascular side effects (e.g., arrhythmias, ischemic effects)	3
Potential effects on hemostasis (e.g., thrombotic effects, bleeding risk)	3
Potential effects on food intake (e.g., loss of appetite, stomach complaints, change of taste)	3
Important drug-disease interactions (e.g., exacerbation of heart failure)	3
Effects on the quality of life*	4
**CONVENIENCE OF USE**	
The convenience of use for older persons (dosage form and packaging)	3
Information should be available about dosing instructions*	4
Aspects related to medication error (invented name and pack design, suitability of a device to avoid mistakes in dosing)*	4

1– Items described in the ICH E7 guideline.

2– Items described in the questions and answers document, supplement to the ICH E7 guideline.

3– Items described in the Dutch formulary.

4– Items suggested by the respondents in first questionnaire of the present study.

The items in the questionnaire were grouped into five themes, namely, pharmacokinetics, efficacy, and safety of medicines in older people, representation of older participants in clinical trials, and the convenience of medication use for older patients.

The respondents indicated, on a Likert scale from 1 (not needed) to 10 (obligatory), whether they thought that this information should be available prior to market approval of a new medicine. A ‘no opinion’ option was available. Space was left for the respondents to make comments on the questionnaire or suggestions for items that should be included. Space was left for the respondents to make comments on the questionnaire or suggestions for items that should be included. The relevance of the suggestions made by the respondents was investigated by means of a second questionnaire (File S2), sent to all respondents. This second questionnaire contained the additional items suggested by the respondents as well as control items from the initial questionnaire, in order to test response consistency.

The questionnaires were sent by e-mail and respondents were allowed 2 weeks to fill in and return the questionnaire by e-mail or post of fax. Initial non-responders were sent a reminder by email after these 2 weeks.

### Data Analysis and Statistics

Median and 10^th^ and 90^th^ percentiles were calculated for the responses to the questionnaire and divided in three categories, based on the median group score: 1) ‘necessary information’, for a median score between 7.5 and 10; 2) ‘uncertain’, for a median score between 3.5 and 7.5; and 3) ‘unnecessary information’, for a median score between 1 and 3.5 [11]. Tenth and 90^th^ percentiles are reported because they were considered to reflect the group opinion better than the range, which includes outlying single opinions.

The Mann-Whitney U test was used to examine differences between clinical respondents (physicians and pharmacists) and non-clinical respondents (regulators, pharmaceutical industry, ethicists, and scientists). The Wilcoxon signed ranks test was used to examine differences in the scores on the control items between the first and the second questionnaires. Eleven items from the initial questionnaire were used as control items in the second questionnaire. The Wilcoxon signed ranks test showed no significant differences in these items, except for the item on the convenience of use. The median score was 8.0 (10^th^ percentile 5.8, 90^th^ percentile 10.0) in the first questionnaire and 8.5 (10^th^ percentile 3.0; 90^th^ percentile 10.0) in the second (p value 0.04). Based on this response consistency, for the analyses, the results from the first questionnaire were used, together with the results on the new items from the second questionnaire. Statistical analysis was performed with SPSS version 20.0 (IBM SPSS Inc., Chicago, IL, USA).

## Results

### Characteristics of Respondents

The characteristics of the respondents are given in table 2. Of the 43 respondents included, 37 (86%) returned the initial questionnaire and 31 of these respondents (84%) returned the second questionnaire. All returned questionnaires were analysed. Not all respondents completed all items: six scores were missing and the ‘no opinion’ box in the initial questionnaire was checked five times. All items of the second questionnaire were completed; the ‘no opinion’ box was checked four times.

**Table 2:** Gender, specialty, and working country of the respondents.

Variable	Category		Included respondents (n (%))	Initial questionnaire: respondents (n (%))	Additional questionnaire: respondents (n (%))
	**Total**		**43**	**37**	**31**
Gender	Male		25 (58)	21 (57)	17 (55)
	Female		18 (42)	16 (43)	14 (45)
Specialty	Clinical	**Total**	**26**	**23**	**21**
		Geriatrician	23	20	18
		Other physician*	2	2	2
		Pharmacist	1	1	1
	Non-clinical	**Total**	**17**	**14**	**10**
		Regulator	10	8	6
		Pharma group	3	3	2
		Ethicist	2	1	0
		Clinical researcher	2	2	2
Country	Austria		1	1	1
	Belgium		2	2	2
	Czech Republic		2	2	2
	Denmark		1	1	1
	Estonia		1	1	1
	Finland		1	1	1
	France		6	5	5
	Germany		4	3	0
	Greece		1	1	1
	Italy		3	3	3
	Netherlands		4	4	3
	Norway		2	1	1
	Poland		2	2	1
	Spain		2	2	2
	Sweden		1	1	1
	Switzerland		3	2	2
	United Kingdom		7	5	4

* Internist, nursing home physician.

Fourteen of the 37 respondents (38%) made comments about or suggested additions to the first questionnaire. Some expressed having difficulty with the generalising nature of the Likert scale, and one respondent indicated that this was the reason why some items were scored 9 instead of 10. Some respondents indicated that some questions were not clearly formulated and other comments revealed that the head of the column in the first survey (“information should be available about…”) was not taken into account in all items.

Six of the 31 respondents (19%) made comments about the second questionnaire, but these were more about personal opinions (“In my opinion, it is as important to have the pharmacokinetic studies in single- and multiple-dose studies, given the different body composition in older persons.” or “Rather than quality of life, which is often an insensitive and difficult to interpret outcome, the influence of new therapeutic agent on Daily Life Activities and Physical Functioning is relevant.”) than about poorly formulated questions.

### Questionnaire Themes

#### Representation of older participants in clinical trials

**Figure 1:**
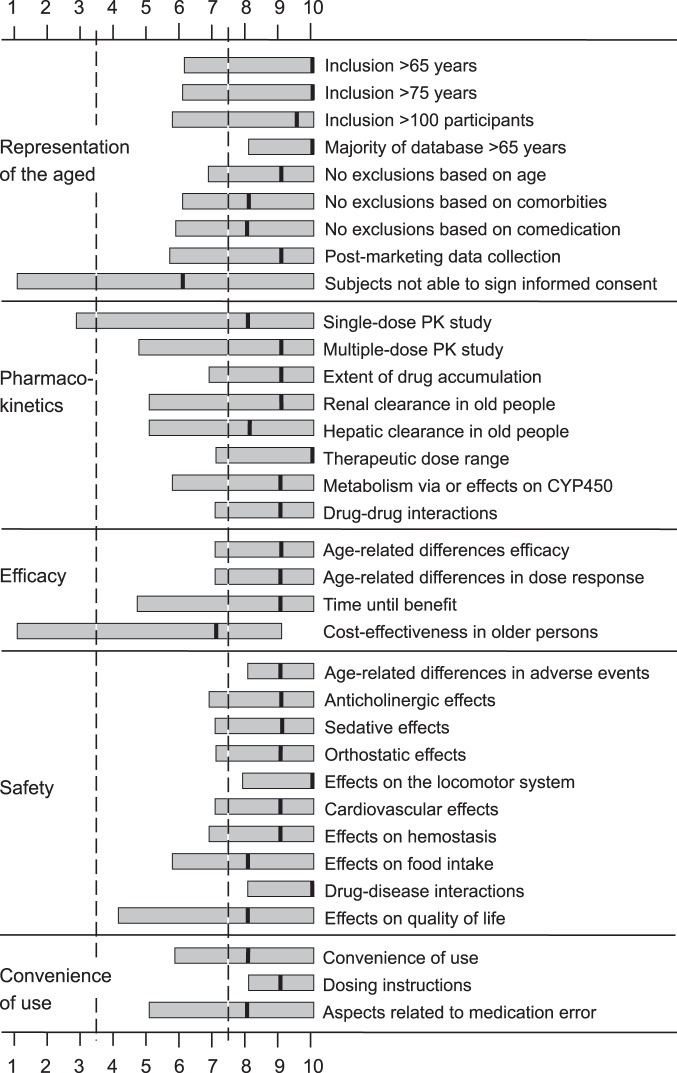
Respondents’ scores on the 34 items in both questionnaires. The respondents indicated, on a Likert scale from 1 (not needed) to 10 (obligatory) (X axis), whether they thought that information on the topic (Y axis) should be available prior to market approval of a new medicine. Median and 10^th^ and 90^th^ percentiles are shown for the responses to the questionnaires and divided in three categories, based on the median group score: 1) ‘necessary information’, for a median score between 7.5 and 10; 2) ‘uncertain’, for a median score between 3.5 and 7.5; and 3) ‘unnecessary information’, for a median score between 1 and 3.5.

Figure 1 shows the overall median scores and the 10^th^ and 90^th^ percentiles of the respondents on the items in the first and second questionnaire.

Information about the representation of older people in clinical trials was considered necessary (median >7.5), except for the information about the number of subjects being included in the clinical program who were not able to sign informed consent themselves (median 6.0) (Figure 1). All respondents considered information about the majority of the included patients being >65 years regarding diseases characteristically associated with aging to be essential (median score >8.0).

The respondents commented extensively on the item stating that at least 100 patients aged 65 years or older should be included in the phase III studies if a drug is indicated for a disease not unique to, but common in, old age. It was felt that, in practice, this would mean that no more than 100 patients would be included. Respondents commented that the population studied should reflect the target population. With rare diseases, it might be sufficient to recruit fewer older participants.

### Pharmacokinetics in Older People

All information about pharmacokinetics in older individuals was considered essential (median >7.5) (Figure 1). More respondents considered information about multiple-dose pharmacokinetic studies in older patients to be more important (median 9; 10^th^ percentile 5.5) than information on single-dose pharmacokinetic studies in older patients (median 8; 10^th^ percentile 3.6; p<0.05). Information about the renal clearance of a drug was considered more important (median 9; 10^th^ percentile 5.6) than information about the hepatic clearance of the drug (median 8; 10^th^ percentile 5.0; p<0.05). Most respondents considered information about the therapeutic dose range to be obligatory (median 10; 10^th^ percentile 7.0).

#### Efficacy of medicines in older people

The respondents considered information about age-related differences in efficacy and dose-response to be essential (median 9.0; 10^th^ percentile 7.0) (Figure 1). Information about the time until benefit in older people was also considered necessary, but the range of responses was wider (median 9.0; 10^th^ percentile 5.0). Respondents were less certain about the importance of information on cost-effectiveness in older people (median 7.0; 10^th^ percentile 1.0).

#### Safety of medicines in older people

All respondents considered information about age-related differences in adverse events (median 9.0; 10^th^ percentile 8.0), effects on the locomotor system (median 10.0; 10^th^ percentile 8.0), and drug-disease interactions (median 10.0; 10^th^ percentile 8.0) to be necessary (Figure 1), and most respondents considered information about how a drug affects food intake (median 8.0; 10^th^ percentile 6.0) and quality of life (median 8.0; 10^th^ percentile 4.0) to be important, but scores showed more variation.

#### Convenience of use for older patients

Most respondents considered information about the convenience of medication use in older people (i.e. about dosing forms and packaging) (median 8.0; 10^th^ percentile 5.0) and information about aspects related to medication errors, such as the pack name and design and the suitability of a device to avoid mistakes in dosing (median 8.0; 10^th^ percentile 4.0), to be important (Figure 1). All respondents agreed that information on dosing instructions was essential (median 9.0; 10^th^ percentile 8.0).

### Clinical vs. Non-clinical Professionals

On most items, the opinions of the non-clinical respondents, i.e. regulators, professionals from the pharmaceutical industry, an ethicist, and a researcher, were not significantly different from those of the clinical respondents (physicians and pharmacists) (Table 3). However, while both clinical and non-clinical respondents considered information about the inclusion of patients aged 65 years or older important, the non-clinical respondents considered information about the inclusion of patients aged 75 years and older less important than did the clinical respondents (median 8.0, interpercentile range 5.5–10.0 versus median 10.0, interpercentile range 8.3–10.0, respectively; p<0.05). The same was true for information about the time until benefit for drugs for chronic use (clinical respondents median 9.0, interpercentile range 6.0–10.0 versus non-clinical respondents median 7.0, interpercentile range 1.4–9.6; p<0.05). The non-clinical respondents considered information about anticholinergic effects to be less important than did the clinical professionals (median 8.0, interpercentile range 4.5–10.0 and median 10.0, interpercentile range. 7.4–10.0, respectively; p<0.05). This was also the case for information about drug-disease interactions, such as exacerbation of heart failure caused by a medicine prescribed for a different indication (non-clinical respondents median 9.0, interpercentile range 7.5–10.0 versus clinical respondents median 10.0, interpercentile range 8.4–10.0, respectively; p<0.05).

**Table 3:** Differences in scores between clinical and non-clinical respondents.

Item	Clinical respondents median (10^th^, 90^th^ percentile)	Non-clinical respondents median (10^th^, 90^th^ percentile)	p value
**REPRESENTATION OF THE AGED**			
Inclusion >65 years	10.0 (6.4–10.0)	8.5 (6.0–10.0)	0.16
Inclusion >75 years	**10.0 (8.3–10.0)**	**8.0 (5.5–10.0)**	**<0.05**
Inclusion >100 patients >65 years	9.0 (6.0–10.0)	9.0 (2.6–10.0)	0.77
Majority of database >65 years	10.0 (8.0–10.0)	9.5 (7.0–10.0)	0.63
No exclusions based on age	9.0 (8.0–10.0)	8.5 (6.0–10.0)	0.14
No exclusions based on comorbidities	8.0 (6.8–10.0)	7.5 (5.0–10.0)	0.09
No exclusions based on comedication	8.0 (6.4–10.0)	8.5 (5.0–10.0)	0.48
Post-marketing data collection	9.0 (5.2–10.0)	10.0 (4.0–10.0)	0.08
Subjects not able to sign informed consent form*	8.0 (1.0–10.0)	5.0 (1.1–8.9)	0.17
**PHARMACOKINETICS**			
Single-dose PK study	9.0 (3.2–10.0)	7.5 (2.0–10.0)	0.29
Multiple-dose PK study	9.5 (5.3–10.0)	8.0 (2.5–10.0)	0.09
Extent of drug accumulation	9.0 (7.0–10.0)	8.0 (6.0–10.0)	0.17
Renal clearance in old people	10.0 (6.0–10.0)	8.0 (1.5–10.0)	0.08
Hepatic clearance in old people	8.0 (6.0–10.0)	8.0 (1.5–10.0)	0.34
Therapeutic dose range	10.0 (7.3–10.0)	9.5 (5.5–10.0)	0.33
Metabolism via or effects on CYP450	9.0 (5.4–10.0)	9.0 (5.4–10.0)	0.52
Drug-drug interactions	10.0 (7.4–10.0)	9.0 (4.5–10.0)	0.10
**EFFICACY**			
Age-related differences in efficacy	9.0 (8.0–10.0)	8.5 (6.0–10.0)	0.18
Age-related differences in dose-response	9.0 (8.0–10.0)	9.0 (6.0–10.0)	0.25
Time until benefit	**9.0 (6.0–10.0)**	**7.0 (1.4–9.6)**	**<0.05**
Cost-effectiveness in older persons*	8.0 (5.0–9.0)	2.0 (1.0–9.2)	0.11
**SAFETY**			
Age-related differences in adverse events	9.0 (8.0–10.0)	9.5 (8.0–10.0)	0.82
Anticholinergic effects	**10.0 (7.4–10.0)**	**8.0 (4.5–10.0)**	**<0.05**
Sedative effects	10.0 (8.0–10.0)	8.5 (6.0–10.0)	0.09
Orthostatic effects	10.0 (7.4–10.0)	8.0 (6.0–10.0)	0.07
Effects on the locomotor system	10.0 (8.4–10.0)	9.0 (4.5–10.0)	0.15
Cardiovascular effects	9.0 (8.0–10.0)	9.5 (7.0–10.0)	0.77
Effects on hemostasis	9.0 (7.4–10.0)	9.5 (5.0–10.0)	0.79
Effects on food intake	9.0 (6.0–10.0)	7.5 (5.0–10.0)	0.06
Drug-disease interactions	**10.0 (8.4–10.0)**	**9.0 (7.5–10.0)**	**<0.05**
Effects on quality of life*	8.0 (6.0–10.0)	6.0 (2.1–9.9)	0.08
**CONVENIENCE OF USE**			
Convenience of use	**9.0 (6.4–10.0)**	**7.0 (5.0–10.0)**	**<0.05**
Dosing instructions*	9.0 (8.0–10.0)	9.5 (7.1–10.0)	0.87
Aspects related to medication error*	8.0 (5.0–9.9)	7.0 (5.1–10.0)	0.98

* Items of the second questionnaire.

Information about the convenience of use was considered more important by the clinical respondents (median 9.0, interpercentile range 6.4–10.0) than by the non-clinical respondents (median 7.0, interpercentile range 5.0–10.0; p<0.05).

Information about five of the ten items rated by the clinical respondents as being most important (10^th^ percentile >8.0), namely, information about the sedative, cardiovascular, locomotor effects, drug–disease interactions, and dosing instructions for older patients, is not described in the ICH E7 guideline or in the Q&A document.

## Discussion

This study investigated which information about older patients clinical and non-clinical professionals consider should be included in the registration dossier for a new medicine. All respondents thought providing information about older people is important and considered information about the inclusion of older participants in the clinical development program obligatory. This reflects the current discussion about older people still being underrepresented in studies of many diseases associated with aging, such as acute coronary syndrome [4], heart failure [12] and Parkinson’s disease [13] even though the Food and Drug Administration, the European Medicines Agency, and clinicians have stressed the importance of including more older participants in clinical trials [3], [5], [7], [8], [14], [15].

Information about the therapeutic dose range was considered very important, as was the information about age-related differences in adverse events, drug-disease interactions, and dosing instructions. The first two items are included in the ICH E7 guideline. A previous study showed that information about age-related differences in adverse events was included in 40% (21/53) of the SmPCs and in 74% (39/53) of the European public assessment reports (EPARs), the public surrogate of the pre-authorisation dossier [16]. Information about the therapeutic dose range could be found in 51% (27/53) of the SmPCs and in 89% (47/53) of the EPARs. Thus, the information considered very important by health care professionals appears to be covered in pre-authorisation dossiers.

The stipulation that at least 100 participants aged >65 years should be included in trials comes from the ICH E7 guideline, which also states that the composition of the study population should reflect that of the general population [7]. In the Q&A document, the EMA emphasized that more than 100 patients is usually appropriate in phase II and III studies [8]. A study on the number of older patients included in the phase II and phase III trials of recently registered drugs showed that about 15% of participants included in trials for two medicines for diabetes mellitus type II (both marketed since 2009) were aged 65 years and older, with a minimum of 108 and a maximum of 887 older participants (Beers E et al., unpublished data); about 1% of the study population was aged 75 years and older (n = 55–83). This small proportion of older adults is striking given that most people with diabetes in developed countries are 65 years or older [17].

### Inter-Professional Differences

A striking finding was that the non-clinical respondents considered information about the inclusion of participants older than 75 years significantly less important than did the clinical respondents. Even so, the non-clinical respondents considered it more important to mention information about the inclusion of patients older than 65 years than information about the inclusion of patients aged 75 years and older, while the reverse was true for the clinical respondents. As is a commonly accepted theory, the risk of frailty increases with age, and frailty is accompanied by a higher risk of adverse outcomes [18]. Therefore, it is perhaps to be expected that the clinical respondents considered information about this age range important. The non-clinical respondents were also less concerned than the clinical respondents about the inclusion of information about the drug’s potential to cause anticholinergic effects. This is surprising because drugs with anticholinergic potential are regarded inappropriate in the older population; both the American Beers list [19] and the European START-STOPP criteria [20] recommend that this group of medicines should be avoided. As the anticholinergic load correlates with the severity of adverse events [21], it is important to have this information available in daily clinical practice.

The non-clinical respondents also considered the convenience of drug use, such as dosage form and packaging, to be less important, although several studies have shown that difficulties with drug dosing and packaging are common, especially among older patients, and give rise to problems ranging from mild inconvenience to serious complications [22].

Information about the time until benefit in old persons for medicines intended for chronic use was considered significantly more important by the clinical. It is perhaps to be expected that the non-clinical professionals attached less importance to the concept of time until benefit, since they are aware that clinical trial duration mostly is relatively short, resulting in the concept of time until benefit being difficult to use, especially for a subgroup of, sometimes underrepresented, older patients [23].

These results are consistent with the professional differences recently found by Crome *et al*., in which geriatricians, general practitioners, nurses, ethicists, clinical researchers as well as pharmacologists and pharmacists working in the pharmaceutical industry from nine European countries were asked for their opinions on the exclusion of older people [14]. Geriatricians as a group were most likely to agree with the statement that older people are under-represented in clinical trials, that older people were disadvantaged by this under-representation and that, as a result, healthcare professionals experience difficulties in prescribing medication to older patients.

### Study Strengths and Limitations

The focus of this study was on the information about older patients for the rational prescription of medicines from a healthcare professional’s point of view. Although the ICH E7 guideline and its supplementary Q&A document have the same goal, their focus is on legislative aspects [7], [8]. The current study suggests that the ICH E7 guideline is not yet optimal.

A strength of this study is that the respondents were given the opportunity to comment on the questionnaire, which resulted in the inclusion of five new items in the second questionnaire. The response consistency between the two questionnaires was considered adequate. Further, it was explicitly stated that information should be *needed* to know, rather than *nice* to know, in order to filter out individual wishes [24]. The respondents came from several European countries, thereby covering inter-country differences, as was also seen in a recent study investigating the professional views on the exclusion of older people from clinical trials in nine European countries [14].

A potential source of bias is that the respondents willing to participate in this study might have been particularly concerned about the availability of information on older patients. Moreover, not all the professional groups were represented in satisfying number and this was especially the case for the pharmaceutical industry and for the sole pharmacist. This problem was partly addressed by creating groups of clinical and non-clinical professionals. Another limitation is that no general practitioners were selected, although in several countries, they are responsible for the prescription of medication to older patients. Furthermore, although we originally intended to include five representatives from individual countries, the response rate differed greatly between the countries, a difficulty recognised earlier by Crome *et al.* [14].

The limitations of a questionnaire are clear: it can be difficult to award answers a score from 1 to 10 and answers may depend on the therapeutic indication or on the behaviour of a drug in the body. This might have influenced the answers given to the pharmacokinetic items and the items on the representation of the older individuals in particular, with the respondents giving lower scores as a reflection of this uncertainty. It was apparent that not all respondents took the column heading (“information should be available about…”) into account, but this issue was resolved in the second questionnaire. However, since the control items were consistently answered in the two questionnaires, the phrasing may have not played a major role.

### Next Steps

As mentioned above, the results suggest that the ICH and/or the EMA could improve the information base about the rational use of medicines in the older population, especially with regard to safety aspects. The ICH E7 guideline does state that there should be an evaluation of age-related differences in adverse events, but it has become clear that clinical professionals need more specific information about the safety aspects of medicines, such as sedative, cardiovascular, and locomotor effects, as well as information about drug-disease interactions and dosing instructions for older patients. Crome *et al*. found that respondents from pharmaceutical industries were least likely to respond that clinical trial regulation needs to be amended, although 56% still did so. The present arrangements for the inclusion of older participants, such as the ICH E7, were considered unsatisfactory by the majority of the geriatricians and nurses [14].

One of the limitations of the present study was the low number of representatives of the groups of pharmacists and pharmaceutical industries and the lack of inclusion of general practitioners. These issues should be addressed in future research as well as in policy making. Fortunately, several steps have already been taken, such as the institution of the Geriatric Expert Group by the EMA, which involves different clinical practitioners, as well as the PREDICT Charter that aims to promote the inclusion of older people in clinical trials, to prevent discrimination on the base of age and to defend the rights of older people in clinical trials [25].

### Conclusions

All respondents thought providing information about older people is important. A number of items considered most important are currently not included in the ICH E7 guideline or its supplement, the Q&A document, namely, information about effects on the locomotor system, drug-disease interactions, and dosing instructions. This suggests that the ICH E7 guideline should be optimised, moreover since the views of the regulatory authorities and the pharmaceutical industry differ from those of the clinical practitioners on the relevance of information in the pre-authorisation dossier of new medicines. Since the latter are the people who have to advice on or to prescribe medication to frail, older patients, more practical information should be available. The pre-authorisation dossier would seem to be the appropriate document for this information, because it is used to prepare the EPAR as well as in the SmPC and PI. It is important that all stakeholders participate in efforts to improve the availability of information about older people to clinical practitioners.

## Supporting Information

File S1 (DOC)

File S2 (DOC)
