# Supplementary material for: The Views of Healthcare Professionals, Drug Developers and Regulators on Information about Older People Needed for Rational Drug Prescription
Source: PLoS One. 2013 Aug 16;8(8):e72060. doi: 10.1371/journal.pone.0072060 (PMC3745417; doi:10.1371/journal.pone.0072060)
Supplement: File S1 — (DOC) [file pone.0072060.s001.doc]

**Supporting file A. First questionnaire containing 29 items**

|  | **ITEMS** | **not needed** | | | | | **obligatory** | | | | | **no opinion** |
| --- | --- | --- | --- | --- | --- | --- | --- | --- | --- | --- | --- | --- |
|  | **Information should be available about:** | 1 | 2 | 3 | 4 | 5 | 6 | 7 | 8 | 9 | 10 |  |
| 1 | Inclusion of patients >65 years in phase III studies |  |  |  |  |  |  |  |  |  |  |  |
| 2 | Inclusion of patients >75 years in phase III studies |  |  |  |  |  |  |  |  |  |  |  |
| 3 | For drugs used in diseases not unique for, but present in, old persons: inclusion of at least 100 patients >65 years in the phase III studies |  |  |  |  |  |  |  |  |  |  |  |
| 4 | For drugs used in diseases characteristically associated with aging (e.g. Alzheimer's disease): the majority of the clinical database consists of geriatric patients |  |  |  |  |  |  |  |  |  |  |  |
| 5 | No exclusion of patients on the basis of an upper age cut-off |  |  |  |  |  |  |  |  |  |  |  |
| 6 | No exclusion based on concomitant medical conditions common in old persons (e.g. cardiovascular disease, diabetes, dementia) |  |  |  |  |  |  |  |  |  |  |  |
| 7 | No exclusion based on concomitant treatment with drugs commonly prescribed for old persons |  |  |  |  |  |  |  |  |  |  |  |
| 8 | Age-related differences in efficacy |  |  |  |  |  |  |  |  |  |  |  |
| 9 | Age-related differences in dose-response |  |  |  |  |  |  |  |  |  |  |  |
| 10 | Age-related differences in adverse events |  |  |  |  |  |  |  |  |  |  |  |
| 11 | A single-dose pharmacokinetic study in young versus old persons |  |  |  |  |  |  |  |  |  |  |  |
| 12 | A multiple-dose pharmacokinetic study in young versus old persons, if there are age-related differences in pharmacokinetics |  |  |  |  |  |  |  |  |  |  |  |
| 13 | The extent of drug accumulation in old persons |  |  |  |  |  |  |  |  |  |  |  |
| 14 | The extent of renal clearance of the active substances (i.e. parent compound and/ or metabolites) in old persons |  |  |  |  |  |  |  |  |  |  |  |
| 15 | The extent of hepatic clearance of the active substances (i.e. parent compound and/ or metabolites) in old persons |  |  |  |  |  |  |  |  |  |  |  |
| 16 | Potential anticholinergic effects (e.g. cognitive decline, delirium, blurred vision, urine retention) |  |  |  |  |  |  |  |  |  |  |  |
| 17 | Potential sedative effects |  |  |  |  |  |  |  |  |  |  |  |
| 18 | Potential orthostatic effects |  |  |  |  |  |  |  |  |  |  |  |
| 19 | Potential effects on the locomotor system (e.g. decline of mobility, increased incidence of falls) |  |  |  |  |  |  |  |  |  |  |  |
| 20 | Potential cardiovascular side effects (e.g. arrhythmias, ischemic effects) |  |  |  |  |  |  |  |  |  |  |  |
| 21 | Potential effects on hemostasis (e.g. thrombotic effects, bleeding risk) |  |  |  |  |  |  |  |  |  |  |  |
| 22 | Potential effects on food intake (e.g. loss of appetite, stomach complaints, change of taste) |  |  |  |  |  |  |  |  |  |  |  |
| 23 | The therapeutic dose range of the drug |  |  |  |  |  |  |  |  |  |  |  |
| 24 | The extent of metabolism via or effects on specified CYP450 enzymes |  |  |  |  |  |  |  |  |  |  |  |
| 25 | Potential drug-drug interactions, if the therapeutic range of the drug or likely concomitant drugs is narrow, and the likelihood of the concomitant therapy is great |  |  |  |  |  |  |  |  |  |  |  |
| 26 | Important drug-disease interactions (e.g. exacerbation of heart failure) |  |  |  |  |  |  |  |  |  |  |  |
| 27 | If the medicinal product is indicated for a chronic condition: time until benefit in old persons |  |  |  |  |  |  |  |  |  |  |  |
| 28 | The convenience of use for older persons (dosage form and packaging) |  |  |  |  |  |  |  |  |  |  |  |
| 29 | The post-marketing data collection in geriatric patients is specified in the Risk Management Plan |  |  |  |  |  |  |  |  |  |  |  |
