# Supplementary material for: The Views of Healthcare Professionals, Drug Developers and Regulators on Information about Older People Needed for Rational Drug Prescription
Source: PLoS One. 2013 Aug 16;8(8):e72060. doi: 10.1371/journal.pone.0072060 (PMC3745417; doi:10.1371/journal.pone.0072060)
Supplement: File S2 — (DOC) [file pone.0072060.s002.doc]

**Supporting file B. Additional questionnaire containing five items suggested by the respondents and 11 control items**

|  | **ITEMS** | **not needed** | | | | | **obligatory** | | | | | **no opinion** |
| --- | --- | --- | --- | --- | --- | --- | --- | --- | --- | --- | --- | --- |
|  |  | 1 | 2 | 3 | 4 | 5 | 6 | 7 | 8 | 9 | 10 |  |
| a | For drugs used in diseases not unique for, but present in, old persons: information should be available about whether at least 100 patients >65 years in the phase III studies have been included |  |  |  |  |  |  |  |  |  |  |  |
| b | Information should be available about a single-dose pharmacokinetic study in young versus old persons |  |  |  |  |  |  |  |  |  |  |  |
| c | Information should be available about the convenience of use for older persons (dosage form and packaging) |  |  |  |  |  |  |  |  |  |  |  |
| d | Information should be available about a multiple-dose pharmacokinetic study in young versus old persons, if there are age-related differences in pharmacokinetics |  |  |  |  |  |  |  |  |  |  |  |
| e | Information should be available about effects on the quality of life |  |  |  |  |  |  |  |  |  |  |  |
| f | Information should be available about the extent of renal clearance of the active substances (i.e. parent compound and/ or metabolites) in old persons |  |  |  |  |  |  |  |  |  |  |  |
| g | Information should be available about the extent of hepatic clearance of the active substances (i.e. parent compound and/ or metabolites) in old persons |  |  |  |  |  |  |  |  |  |  |  |
| h | Information should be available about cost-effectiveness in older persons |  |  |  |  |  |  |  |  |  |  |  |
| i | Information should be available about dosing instructions |  |  |  |  |  |  |  |  |  |  |  |
| j | Information should be available about the extent of metabolism via or effects on specified CYP450 enzymes |  |  |  |  |  |  |  |  |  |  |  |
| k | Information should be available about the extent of drug accumulation in old persons |  |  |  |  |  |  |  |  |  |  |  |
| l | Information should be available about potential sedative effects |  |  |  |  |  |  |  |  |  |  |  |
| m | Information should be available about potential cardiovascular side effects (e.g. arrhythmias, ischemic effects) |  |  |  |  |  |  |  |  |  |  |  |
| n | Information should be available about how many subjects were included in the clinical program, who were not able to sign informed consent form themselves |  |  |  |  |  |  |  |  |  |  |  |
| o | Important drug-disease interactions (e.g. exacerbation of heart failure) |  |  |  |  |  |  |  |  |  |  |  |
| p | Information should be available about aspects related to medication error (invented name and pack design, suitability of a device to avoid mistakes in dosing) |  |  |  |  |  |  |  |  |  |  |  |
